# Timeline of SARS-CoV-2 Transmission in Sabah, Malaysia: Tracking the Molecular Evolution

**DOI:** 10.3390/pathogens12081047

**Published:** 2023-08-15

**Authors:** Krishnan Nair Balakrishnan, Chee Wei Yew, Eric Tzyy Jiann Chong, Sylvia Daim, Nurul Elyani Mohamad, Kenneth Rodrigues, Ping-Chin Lee

**Affiliations:** 1 Biotechnology Research Institute, Universiti Malaysia Sabah, Jalan UMS, Kota Kinabalu 88400, Sabah, Malaysia; krishnannair@ums.edu.my (K.N.B.); cheewei.yew@ums.edu.my (C.W.Y.); eric_ctj@ums.edu.my (E.T.J.C.); elyani.mohamad@ums.edu.my (N.E.M.); kennethr@ums.edu.my (K.R.); 2 Faculty of Medicine and Health Sciences, Universiti Malaysia Sabah, Jalan UMS, Kota Kinabalu 88400, Sabah, Malaysia; sylviadaim@ums.edu.my; 3 Faculty of Science and Natural Resources, Universiti Malaysia Sabah, Jalan UMS, Kota Kinabalu 88400, Sabah, Malaysia

**Keywords:** COVID-19, SARS-CoV-2 genome, Sabah, epidemiology, mutations

## Abstract

Background: The COVID-19 pandemic poses an unprecedented public health challenge in Malaysia. The impact of COVID-19 varies between countries, including geographically divided states within a country. The deadly transmission of COVID-19 has taken a heavy toll in Sabah, Malaysia’s third most populous state, contributing nearly 10% to the recorded national death toll as of 31 December 2022. Although several SARS-CoV-2 genome sequences have been analysed in Malaysia, molecular epidemiology data from Sabah focusing on the diversity and evolution of SARS-CoV-2 variants are still lacking. This study examines the major SARS-CoV-2 variants and emerging mutations from Sabah, the Malaysian Borneo, which is geographically divided from West Malaysia by the South China Sea. Methods: A total of 583 COVID-19 samples were subjected to whole genome sequencing and analysed with an additional 1123 Sabah COVID-19 sequences retrieved from the GISAID EpiCoV consortium. Nextclade and Pangolin were used to classify these sequences according to the clades and lineages. To determine the molecular evolutionary characteristics, Bayesian time-scaled phylogenetic analysis employing the maximum likelihood algorithm was performed on selected SARS-CoV-2 genome sequences, using the Wuhan-Hu-1 sequence as a reference. Results: Sabah was affected starting from the second COVID-19 wave in Malaysia, and the early sequences were classified under the O clade. The clade was gradually replaced during subsequent waves by G, GH, GK and GRA, with the latter being dominant as of December 2022. Phylogenetically, the Delta isolates in this study belong to the three main subclades 21A, 21J and 21I, while Omicron isolates belong to 21M, 21L and 22B. The time-scaled phylogeny suggested that SARS-CoV-2 introduced into Sabah originated from Peninsular Malaysia in early March 2020, and phylodynamic analysis indicated that increased viral spread was observed in early March and declined in late April, followed by an evolutionary stationary phase in June 2020. Conclusion: Continuous molecular epidemiology of SARS-CoV-2 in Sabah will provide a deeper understanding of the emergence and dominance of each variant in the locality, thus facilitating public health intervention measures.

## 1. Introduction

The Coronavirus Disease 2019 (COVID-19) pandemic was announced by the World Health Organization (WHO) in early 2020 [1]. The severe acute respiratory syndrome coronavirus 2 (SARS-CoV-2) has infected more than 600 million people and claimed more than 6 million lives worldwide, placing unprecedented pressure on healthcare and public health systems in many countries [2,3]. After administering vaccines and with a relatively stable number of positive cases, almost all countries are endeavouring to transition to the endemic state of COVID-19. Nevertheless, the continuous emergence of variants threatens the endemic transition, as evidenced by the waves of COVID-19 that occurred among different countries during different periods [4,5,6]. Indeed, SARS-CoV-2 evolves with a mutation rate of approximately 9.8 × 10^−4^ substitutions/site/year, resulting in a high rate of lineage turnover [7,8].

The first SARS-CoV-2 genome sequence was published on 11 January 2020, with GenBank’s accession number MN908947.3 [1]. The SARS-CoV-2 virus is assigned to the B genus of coronaviruses and shares almost 50% and 79% sequence identity with the Middle East Respiratory Syndrome (MERS) and SARS-CoV, respectively [9,10]. The SARS-CoV-2 virus consists of a non-segmented, single-stranded, positive-sense RNA genome protected by an envelope containing a 5′-cap structure and a 3-poly-A tail [11]. The genome size ranges from 29.8 kb to 29.9 kb, with 12 open reading frames (ORFs) encoding 29 proteins with 25 putative non-structural and accessory proteins and four structural proteins [9,12,13]. Two-thirds of the SARS-CoV-2 genome are known to encode non-structural proteins (NSPs) referred to as pp1a and pp1ab polyproteins. The pp1a protein comprises NSP1 to NSP11, while pp1ab corresponds to NSP12 to NSP16. The remaining region encodes four structural proteins known as the spike (S), membrane (M), envelope (E) and nucleocapsid (N), and all functions of these proteins have been previously described [14,15]. Several mutations have been identified in different genes of SARS-CoV-2, and among the significant mutations reported are in S, NSP1, NSP2, NSP3, NSP12, NSP15 and ORF8 [12,16,17]. Among these, S protein, NSP12 RNA-dependent RNA polymerase (RdRp), NSP5 main protease (Mpro), and NSP3 papain-like protease (PLpro) were the prime targets during the development of antiviral agents. The currently available antivirals approved by the US Food and Drug Administration (FDA) or Emergency Use Authorization (EUA) for the treatment of COVID-19 have primarily targeted these proteins [18,19].

Intensive genomic surveillance from different regions was performed simultaneously using next-generation sequencing (NGS) technology to determine the circulating variants. Generating whole genome sequences (WGS) contributes extensively to monitoring the viral spread and mutations that could alter the antigenic properties of the virus. As of February 2023, more than 14 million SARS-CoV-2 WGS have been deposited in the Global Initiative on Sharing All Influenza Data (GISAID) EpiCoV, a publicly accessible database. The availability of massive SARS-CoV-2 genome sequences in public databases enables researchers worldwide to monitor and track the transmission of variants and the occurrence of viral genome mutations within and between countries, continents, and populations. To a greater extent, a nation could recognize a red flag ahead of time to prevent and/or respond to any variant emerging from other parts of the world. To monitor the SARS-CoV-2 evolution, WHO has defined the emerging variants’ status into two categories: Variants of Concern (VOCs) and Variants of Interest (VOIs) based on emerging scientific evidence. VOIs describe a variant with the potential to cause significant community transmission involving multiple COVID-19 clusters. With specific genetic markers associated with the changes in receptor binding sites, VOIs can affect transmission rates and disease severity, reduce the ability to neutralize antibodies from previous vaccinations and warrant continuous monitoring over time [20]. As of December 2022, no circulating variant has been classified as a VOI. However, previously circulating VOIs were Kappa, Lambda, Zeta, Theta, Mu, Eta, Epsilon and Iota. VOCs are variants that meet all the criteria of VOI and have also been shown to increase virulence, transmissibility, disease severity and/or decreases in efficacy of readily available therapeutics, vaccines and diagnostics. Omicron is the only VOC in current circulation, while previous VOCs included Alpha, Beta, Gamma and Delta [20]. In addition, NextStrain, GISAID, and Phylogenetics Assignment of Named Global Outbreak Lineage (Pango lineage) were used to distinguish the genetic lineages of SARS-CoV-2 at the local and global levels. According to the GISAID classification system, which is based on marker mutation, the variants can be distributed into eleven clades starting from L and S. The early split L belongs to the Wuhan reference strain, and further evolution of L split into V and G. Over time, G split into GH, GR, GV and GK. GR then evolved into GRY and GRA as the current dominant clade.

Additionally, clade O grouped all unclassified sequences of SARS-CoV-2. Next, in early 2020, the Pango nomenclature was developed, which differs from other nomenclature systems in generating the epidemiological relevance of their lineages integrated with information on the geographic and genetic composition of SARS-CoV-2 [8,21]. The main advantage presented by the Pango nomenclature system was the ability to analyse complete or near-complete genome sequences of SARS-CoV-2 with high genome coverage, thereby offering reliable evidence of outbreak cluster, facilitating tracking and monitoring of emerging variants locally and globally [21]. Compared to WHO and GISAID, the Pango lineage uses alphabetic prefixes and numeric suffixes for lineage designation. For instance, WHO VOCs such as Alpha, Beta, Gamma, Delta and Omicron corresponded to Pango lineages B.1.1.7, B.1.351, P.1, B.1.617.2 and B.1.1.529, respectively. The Nextstrain nomenclature system labels the SARS-CoV-2 lineages based on the accumulated mutations with the “year-letter” strategy. A new major clade will be assigned when the variant reaches a frequency of 20% worldwide [22]. There are 25 Nextstrain clades, and some dominant clades were 21A (Delta), 21J (Delta), 21M (Omicron), 21L (Omicron) and 22B (Omicron) at the end of 2022.

Sabah is the third most populous state (3.4 million or 10.4%) in East Malaysia, forming the northern part of Borneo and separated from West Malaysia by the South China Sea. Sabah is less economically developed than other states in peninsular Malaysia, has rugged topography, and needs more evaluation for healthcare systems. It has diverse indigenous groups living on the periphery of urban and rural areas. The COVID-19 index in Sabah was known to be unsatisfactory in 2020 and 2021, and is considered one of the states seriously affected by SARS-CoV-2 in Malaysia. Several studies have investigated the genetic epidemiology of SARS-CoV-2 from Malaysia and other states; however, there is still a scarcity of genomic data on the virus from Sabah. The status of COVID-19 in Sabah associated with the circulating lineages and clades during different periods is unclear.

Therefore, this study supports the Malaysian government’s efforts to monitor and track the circulating SARS-CoV-2 variants by analysing the data from genomic surveillance in Sabah during all five waves, including the ongoing pandemic. The SARS-CoV-2 genomes from Sabah were sequenced and subjected to extensive analysis, including other available genomic sequences from the GISAID EpiCoV database as of 31 December 2022. This study is the first to report the genetic diversity of SARS-CoV-2 variants and their distinct lineage/clade replacement associated with different timelines (2020–2022) from Sabah, Malaysia.

## 2. Materials and Methods

### 2.1. Sample Acquisition

A total of 661 COVID-19-positive samples from all districts of Sabah from March 2022 to December 2022 were collected for whole viral genome sequencing. The samples were transported in a viral transport medium using an unbroken cold chain and stored at ultra-low temperature conditions until further use. Total genomic RNA was extracted using the Maxwell kit on Promega’s automated platform following standard protocols [23]. Real-time RT-PCR was performed to screen the samples using Viasure SARS-CoV-2 Real-Time PCR Detection Kit (CerTest Biotec SL, Zaragoza, Spain) according to the manufacturer’s recommendations. Samples exhibiting a cycle threshold (Ct) value below 33 were chosen as the primary inclusion criteria for sequencing. Hence, 583 samples were used for library preparation and sequencing.

### 2.2. Epidemiology of Coronavirus Disease 2019 in Sabah

Official data on COVID-19 epidemiology was retrieved from the Ministry of Health (MOH) official GitHub account at https://github.com/MoH-Malaysia/covid19-public (accessed on 20 January 2023), an open-source software containing all relevant information. The official dataset deposited by MOH on the GitHub account was obtained from various government health systems, such as Crisis Preparedness and Response Centre (CPRC) and National Public Health Laboratory, along with the locally developed MySejahtera mobile application for national COVID-19 surveillance [24]. Any missing information was also sourced from the Malaysian government’s official website for COVID-19, known as COVIDNOW. This study collected and analysed data from the early pandemic to 31 December 2022.

### 2.3. Whole Genome Sequencing of SARS-CoV-2

Libraries for SARS-CoV-2 sequencing were generated using Illumina COVIDseq RUO kits (Illumina Inc., USA) following the manufacturer’s instruction. Complementary DNA (cDNA) was prepared from extracted RNA using the random hexamers, followed by viral genome enrichment using two primer pools: C4P1 and C4P2, by polymerase chain reaction (PCR). The PCR products were further subjected to tagmentation and adapter ligation using 8 IDT for Illumina PCR indexes sets 1 to 4. After enrichment, the tagmented amplicons were purified, and 48 sample batches were pooled together per recommended protocols. In addition, a positive control (CPC HT) and negative control (NTC) were also included during the final pooling. The concentration of the pooled libraries was determined using Qubit 4.0 fluorometer (Invitrogen), and all pools were combined to make a final concentration of 4nM using the Illumina pooling calculator. Prior to sequencing, the final pooled library was denatured, neutralized and diluted to a final concentration of 12 pM. The final pooled library was subjected to paired-end sequencing using the SY-410-1003-Illumina-Miseq platform. The read sequences were converted to a FASTQ file using Illumina Basespace’s online FASTQ generation tool. The reads were aligned to the reference COVID-19 genome SARS-CoV-2 Wuhan-Hu-1 reference genome sequence (GenBank accession no. NC_045512.2) followed by variant calling and consensus sequence generation which were performed using Illumina DRAGEN COVID Lineage application.

### 2.4. Reporting

All SARS-CoV-2 genome sequences obtained in this study with a 98.9–99.9% coverage and more than 29,000 bp in length were deposited to the GISAID database. After receiving the accession numbers, the sequences with relevant information were submitted to the centralized reporting centre of the Malaysia Ministry of Health (MOH).

### 2.5. Analysis of SARS-CoV-2 Sequences from Sabah/Phylogenetic and Mutational Analysis

The sequences obtained from this study and other sequences from Sabah collected from March 2020 to December 2022 were subjected to evolutionary analysis using the GISAID EpiCoV database. A total of 1706 sequences of SARS-CoV-2 from Sabah and the selected sequences were further categorized into GISAID clades and Pango lineages using Nextclade and Pangolin software. Additional filtering options were applied to determine the complete genome (>29,000 nucleotides) with high coverage (<1% of undefined bases) for phylogenetic and mutational analysis. This resulted in a dataset of 1354 SARS-CoV-2. Nextstrain v3.0.3 SARS-CoV-2 [22], a workflow to monitor the real-time evolution of pathogens, was used to construct a phylogenetic tree using all 1354 genomic sequences as an input. Briefly, the input sequences and metadata were subjected to default filtering before aligning with the reference sequence using nextalign. After alignment, IQTree v.2 [25] was used to reconstruct the phylogenetic tree, followed by TreeTime [26] to reroot, correct polytomies, prune sequences, and infer and label the internal nodes of the constructed tree. In addition, the workflow deduced the nucleotides at internal nodes and their translation to amino acid modifications with corresponding labelled clades based on predefined mutations. Finally, JSON files were created as the workflow output and then used as input to the Auspice visualization tool, a web-based software for viewing and exploring the details of the phylogenetic tree comprising 1354 genome sequences SARS-CoV-2 in Sabah. Mutation analyses were performed using Nextclade v.1.5.2 (https://clades.nextstrain.org, accessed on 18 February 2023) compared to the wild-type of Wuhan-Hu-1 (accession number: NC_045512.2.). Mutational event diversity targeting nucleotides and amino acids of the Sabah SARS-CoV-2 Spike glycoprotein was carried out using the Nextstrain server [27], a database used to determine the number of mutations occurring at specific sites in the viral genome.

### 2.6. Phylogeographic and Phylodynamic Reconstructions

A total of 657 SARS-CoV-2 genomes were retrieved from the GISAID database (www.gisaid.org, accessed on 15 March 2023), with exact collection dates (before 30 June 2020) to infer the origin time and transmission of SARS-CoV-2 by Bayesian phylodynamic approaches. Phylogenetic analysis was performed using the maximum likelihood (ML) method based on the time reversible model, and root-to-tip regression was conducted to test the temporal signal, and the effect was evaluated with R square calculated using TempEst v1.5.3 software [28]. Root-to-tip regression analysis showed a positive correlation between sampling time and genetic distance, so the sequences were subjected to subsequent molecular clock calibration. BEAST/BEAGLE v2.5 software was used to reconstruct the evolutionary dynamics of SARS-CoV-2 using the HKY nucleotide substitution model with a strict molecular clock and assumed constant evolutionary rates through Markov chain Monte Carlo (MCMC) [29]. Bayesian analysis was run for 100 million MCMC steps with sampling parameters and trees every 1000 generations. The convergence MCMC chains were viewed using Tracer v1.7.1, and the summary tree was generated using TreeAnnotator v1.10.4 after discarding the first 10% as burn-in. MCC summary tree was visualized using FigTree v1.4.4. The final map was made using QGIS v3.32 to visualize the inferred transmission pattern of SARS-CoV-2 [30].

## 3. Results

### 3.1. The Status of COVID-19 in Sabah

**Figure 1:**
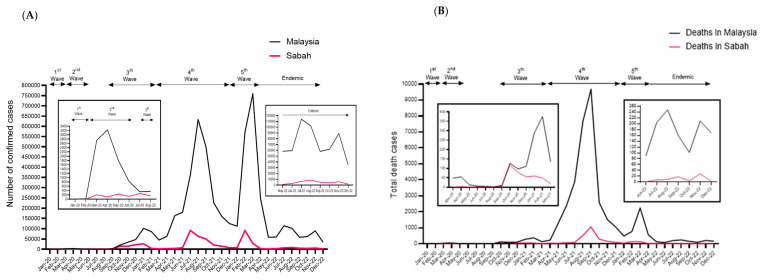
Epidemiology of COVID-19 in Malaysia and Sabah as of 31 December 2022. (**A**) Number of confirmed COVID-19 cases in Malaysia and Sabah. (**B**) Number of confirmed COVID-19 death cases in Malaysia and Sabah. Studied period: March 2020 to December 2022.

The first COVID-19 case in Sabah was registered on 12 March 2020. Since then, 514,981 confirmed COVID-19 cases have been recorded in Sabah as of 31 December 2022. This accounts for 10.2% of the total national COVID-19 cases, covering five epidemic waves since SARS-CoV-2 was first detected in Malaysia. The number of confirmed cases in Sabah from March 2020 to December 2022 is summarized in Figure 1A. With a population of 3.5 million, approximately 15% of the Sabah population has been infected with SARS-CoV-2. On a cautionary note, the percentage may be lower after considering reinfection on an account. Zero cases were reported in January and February 2020 in Sabah, while cases increased drastically during the third wave, with the highest daily cases (26,274) registered in January 2021. Since then, the number of daily cases has remained high, although cases appear to have fluctuated during each successive wave. Notably, 92,112 and 91,435 cases were reported as the highest daily COVID-19 registered in the fourth and fifth waves, respectively, in Sabah. Sabah registered 3195 mortalities, contributing about 9% to the national death toll (36,853) as of December 2022 (Figure 1B). The initial peak in daily deaths was observed in October 2020, when 118 deaths were reported in Sabah. Nevertheless, the highest daily mortality rate was reported during the fourth wave of the COVID-19 outbreak, documenting 529 and 1058 deaths in August and September 2021, respectively. Overall, 511,786 or 99.4% of the infected Sabah population, had recovered from COVID-19. As of 31 December 2022, 1629 SARS-CoV-2 genomic sequences from Sabah, representing 0.3% of Sabah’s COVID-19 cases, have been deposited in the GISAID database.

### 3.2. Analysis of SARS-CoV-2 Sequences from Sabah

**Figure 2:**
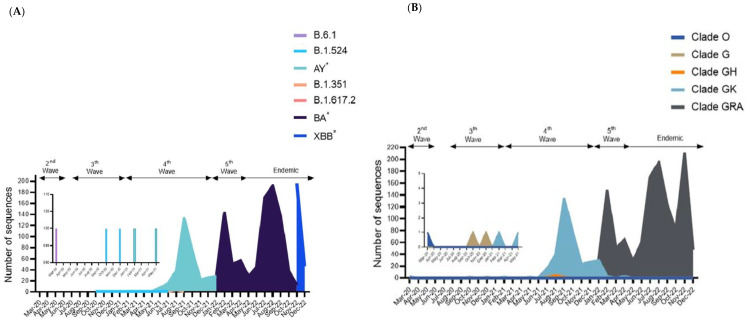
Distribution of lineages among the 1706 genomic sequences in Sabah. (**A**) The number of sequences based on major Pango lineages. (**B**) The number of sequences based on major clades assigned by WHO. Studied period: March 2020 to December 2022. * Indicates all descendent lineages.

The SARS-CoV-2 genome sequences in Sabah can be classified into 111 different Pango lineages (Figure 2A). The most abundant lineages detected in Sabah were BA (57.91%), followed by AY (31.78%), and the remaining lineages were found to have frequencies below 1%. Among the BA lineages of B.1.529 (Omicron), most of the sequences were assigned to XBB (23.51%) over BA.2.3 (13.13%), BA.2 (8.32%), BA.1.1 (3.22%), BA.1 (1.17%) and the rest with frequencies were below 1%. For the AY lineage, the dominant was AY.76 (8.44%), followed by AY.23 (7.80%), AY.59 (1.47%) and AY.24 (1.41). Each specific lineage was dominant during each wave. For instance, the B.6.1 lineage was recorded during the second wave, followed by the B.1.524 lineage during the third wave, the AY and B.1.351 lineages during the fourth wave, the BA lineage during the fifth wave and the XBB lineage during the transition to endemic phase.

Besides Pangolin, all 1706 genomic sequences were classified using GISAID clades, and five distinct major clades were discernible among the Sabah isolates. The dominant clade was GRA (n = 1352), followed by GK (n = 344), GH (6), G (3) and O (1), as shown in Figure 2B. The distribution of these clades varied at different time points of the pandemic in Sabah. Clade GRA continued to dominate during the fifth wave and endemic phase, while GK and GH dominated during the fourth wave. Meanwhile, clades O and G were reported during the second and third waves. There were no records of other clades, including GRY, GR, GV, V and S, in Sabah isolates of SARS-CoV-2.

The genomic sequences of SARS-CoV-2 in Sabah generally fell into the category of VOCs defined by the WHO. The majority of VOC is Omicron (79.25%), followed by Delta (20.16%). The remaining sequences were found to have frequencies below 1% for Beta. No sequences of Alpha and Gamma were recorded in Sabah. The Beta variant appears dominant during the second wave until the Delta and Omicron are replaced during the fourth and fifth waves. In addition, previously circulating VOIs such as Kappa, Theta and Eta were not identified among the Sabah isolates of SARS-CoV-2.

**Figure 3:**
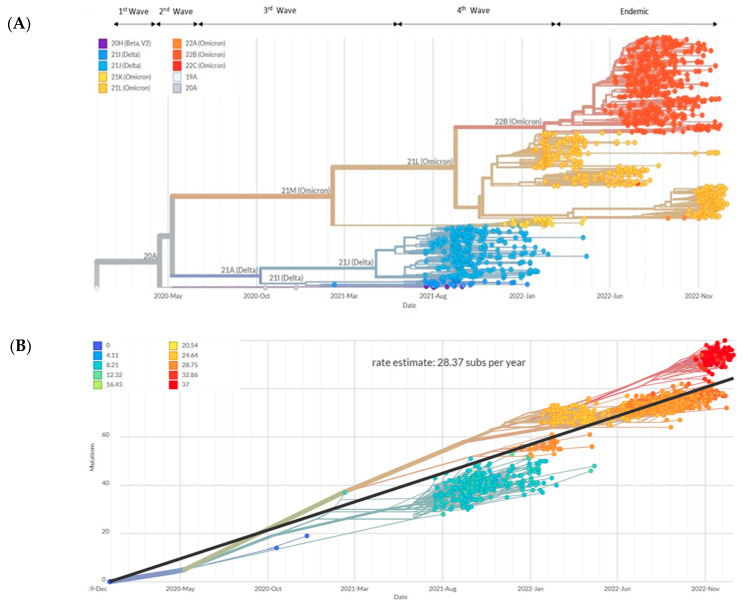
Phylogenetic and phylodynamic of 1354 SARS-CoV-2 genomic sequences in Sabah. (**A**) Phylogenetic tree of all circulating lineages developed through the Nextstrain server (Nextclade tool). (**B**) Phylogenetic tree embedded as a root-to-tip plot, in which the x-axis represents the date of sample collection, and the y-axis represents the number of genome-wide mutations that have occurred since the phylogeny root. The regression line represents the average mutation rate of the SARS-CoV-2 sequences in the tree (28.37 substitutions/year). Studied period: March 2020 to December 2022.

In terms of Nextstrain clade analysis, the constructed tree was constructed using 1354 complete and high-coverage genomic sequences of SARS-CoV-2 sampled in Sabah from March 2020 to December 2022 (Figure 3A). Almost all clades were clustered as expected: clades 21A and 21M diverged from 20A, while 21J and 21I diverged from 21A. Focusing on Omicron, 21L diverged from 21M while 22B diverged from 21L. Overall, the tree follows a similar trend of clades replacement over time, consistent with Pango lineages and GISAID clades. In short, the Beta variant (20H) was detected during the second wave and replaced by Delta variants (21A, 21J, 21I) during the third wave. Then, Omicron variants (21M, 21L, 22B) predominated during the fourth wave and continued to dominate during the fifth and endemic phases (Figure 3A). Root-to-tip regression using molecular clock analysis estimated 28.37 substitutions per year among the SARS-CoV-2 sequences obtained from Sabah (Figure 3B).

**Figure 4:**
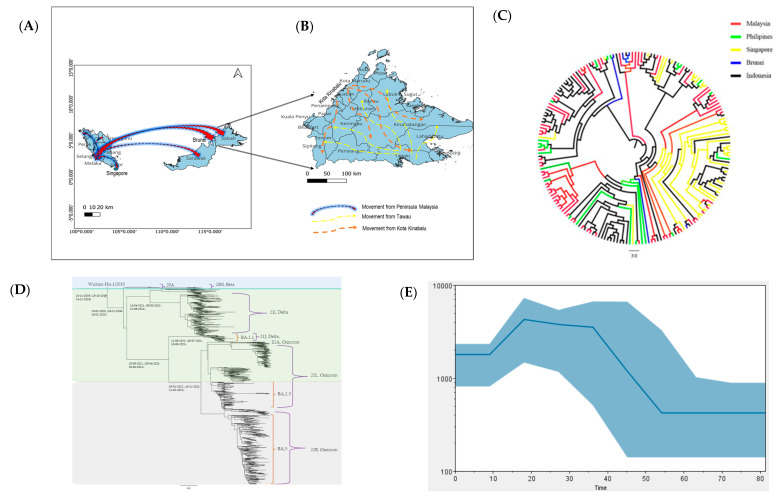
Evolutionary dynamics of SARS-CoV-2 in Sabah. (**A**) Map of transmission pattern of SARS-CoV-2 to Sabah in March 2020 as the first case was reported on 12 March 2020. (**B**) The detailed map of Sabah illustrates the virus transmission pattern with two hotspot zones: Tawau and Kota Kinabalu. (**C**) Time-scaled MCC cladogram based on MCMC analysis of 657 SARS-CoV-2 genomes with exponential growth tree prior. (**D**) MCC tree of SARS-CoV-2 isolated in Sabah. (**E**) Viral population dynamics generated using Bayesian Skyline Plot reconstruction of MCMC analysis. The colour region shows 95% HPD limits, and the blue line represents the median estimate of relative genetic diversity.

According to phylogeographic analysis, the COVID-19 outbreak in Sabah started in the Tawau district (Figure 4A) and the origin of the virus was traced to Peninsula Malaysia, which served as the epicentre of transmission in early March 2020. Subsequently, the virus spread to other regions of Sabah and Kota Kinabalu, becoming the second most infected district and spreading the virus to other parts of Sabah (Figure 4B). Based on the Time-Scaled MCC cladogram, the first Sabah SARS-CoV-2 is shown to have originated in Peninsular Malaysia. However, the basal position is known to have originated in Indonesia. Then, the tree split and contributed to the spread to other countries such as Brunei, the Philippines and Singapore (Figure 4C). The estimated mean of tMRCA of the Sabah SARS-CoV-2 isolate was 94 days (95% high posterior density (HPD): 82.5–154.3), corresponding to the haplotype of the outbreak in Wuhan, China, on 26 November 2019 (HPD 95%: 23 October 2019–24 December 2019) (Figure 4D). The root of the Alpha clade was dated back to 23 January 2020 (HPD 95%: 19 December 2019–18 February 2020) and the Beta and Delta clades to 13 April 2021 (HPD 95%: 5 March 2021–22 May 2021). All Omicron variants in the phylogenetic tree (Figure 4D) were dated back to 3 January 2022 (HPD 95%: 15 December 2021–21 February 2022). The population dynamics of SARS-CoV-2 were plotted using Bayesian Skyline Plot in March 2020, which showed a sigmoidal type of distribution with exponential growth was noticed at the beginning of March 2020 and started to reduce in April and plateaued in May 2020 (Figure 4E).

### 3.3. Mutational Analysis in Sabah SARS-CoV-2 Isolates

**Figure 5:**
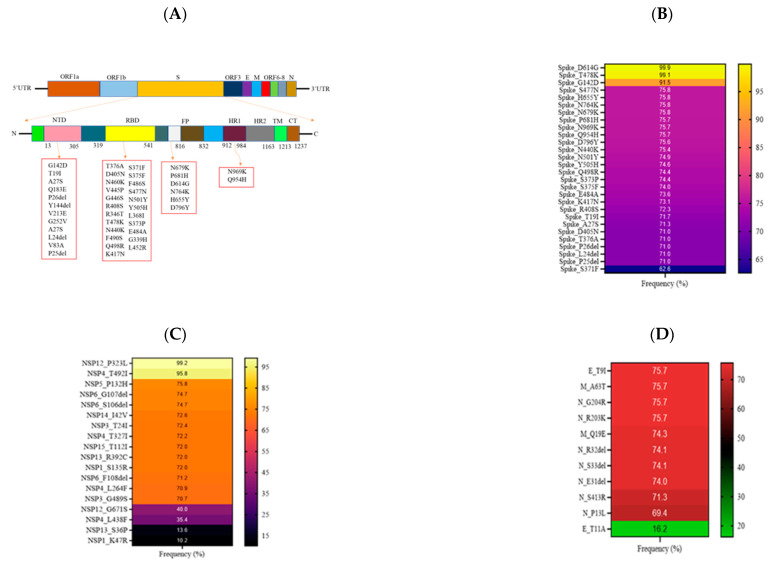
Schematic diagram illustrating the mutational landscape of the SARS-CoV-2 isolates from Sabah. (**A**) Different mutations in several regions in the Spike protein. (**B**) The frequency (%) of significant mutations in Spike protein; (**C**) non-structural proteins; and (**D**) other structural proteins.

The 1354 complete and high coverage genomic sequences of SARS-CoV-2 isolates from Sabah were compared to the reference genome of SARS-CoV-2 isolate from Wuhan, China (hCoV19/Wuhan/WIV04/2019) using the CoVserver tool to examine the occurrence of the mutation. The selected sequences had an average of 56.9 coding mutations per sample (range 4–89) and a median of 61. This study has identified approximately 76 variants in various gene regions with different frequencies. The highest number of mutations occurred in the Spike glycoprotein region (n = 44), followed by Non-structural proteins (NSP, n = 20) and other structural proteins (n = 12). With most mutations in the Spike region, Figure 5A shows the type of mutations associated with the position of Spike protein recorded from Sabah isolates of SARS-CoV-2. The type of mutations that occurred in most Sabah SARS-CoV-2 isolates was captured according to the frequency (%) in spike protein (Figure 5B), NSP (Figure 5C) and other structural proteins (Figure 5D), and the type of mutation that occurred in samples below 10% was not included in the illustration. Analysis of structural proteins showed that the Spike glycoprotein has the largest number of significant mutations, followed by the N gene. On the other hand, the most significant alterations were detected in NSP4, followed by NSP6, in the NSP region. Five mutations were recorded in more than 90% of the samples sequenced, and the top three were occupied by Spike_D614G (99.9%), NSP12_P323L (99.2%) and Spike_T478K (99.1%). The fourth highest mutation occurred in NSP4_492I (95.8%) and the fifth highest in Spike_G142D (91.5%). Most Spike and NSP mutations occurred in 70% of the samples (Figure 5B,C). In addition, the number of mutations in E, M and N gene regions was lower than in Spike and NSP regions. However, most mutations at E, M and N occurred in 70% of the samples sequenced.

Most samples had signature mutations of Omicron lineages in Spike protein, where the mutations present in more than 60% of the samples were reported in this study (Figure 5B). The mutations D405N, R4O8S, P25del, T191, P26del, T376A and S371F were unique to BA.1 and/or BA.2 lineages. Mutations at D614G, G142D, S373P, H655Y, N440K, S375F and K417N were common among all Omicron sublineages. Three mutations (N679K, P681H and N764K) were shared by the BA.1 and BA.2 lineages at the spike polybasic cleavage site, accounting for 75% of the samples. Three substitutions (Q954H, N969K and D796Y) were noted at the Spike C-terminal Domain (CTD) and were shared by all Omicron variants.

More than 10% of the samples had mutations in the ORF1 region (Figure 5C). The NSP1 region had two mutations (S135R and K47R), while no significant mutation was observed in the NSP2 region. NSP3 contained mutations (T24I, G489S) specific to BA.2 lineage, while the NS4 region contained 4 mutations (T327I, T492I, L438F and L264F). The T492I mutation is observed in all Omicron lineages, while the other three mutations are unique to the BA.2 lineage. In NSP5 and NSP14 regions, a single mutation was found at positions P132H and I42V, respectively, where all Omicron lineages shared these common mutations. Two mutations (P323L and G671S) were identified in NSP12, while R392C and S36P mutations were in NSP13. NSP15 contained a single mutation at T112I that belonged to the BA.2 lineage.

A total of 11 significant mutations were observed in the structural proteins of SARS-CoV-2 isolates from Sabah (Figure 5D). Most mutations were observed on the nucleocapsid protein (S413R, S33del, R32del, E31del, P13L, R203K and G204R), shared by all Omicron lineages. Two mutations in the envelope (T91 and T11A) and membrane protein (A63T and Q19E) were common among BA.1 and BA.2 lineages.

## 4. Discussion

Malaysia’s first national COVID-19 case was confirmed on 25 January 2020, forming the country’s first wave of the COVID-19 outbreak. However, Sabah was unaffected during this period, as the first case of COVID-19 in Sabah was reported on 12 March 2020, about two months after the first three cases were detected in Malaysia [31]. The religious gathering known as the Sri Petaling Tabligh cluster caused the second outbreak in the country, and by the end of April 2020, Sabah had recorded 315 confirmed COVID-19 cases with four deaths (https://github.com/MoH-Malaysia/covid19-public, accessed on 20 January 2023). After implementing the government’s movement control order, the number of cases in Sabah remained relatively low until August 2020. However, the number of cases in Sabah contributed approximately 77.8% and 42.3% to the total number of national cases in July and August 2020, respectively. The high number of cases during this period could be due to a high backlog of reported cases [32]. Apart from that, Sabah was tested with large clusters of COVID-19 cases that sparked the country’s third wave of COVID-19 outbreak [33]. This made Sabah the state with the highest COVID-19 cases in September and October 2020, contributing 91% and 71% of the total national cases, respectively. COVID-19 can be asymptomatic, and symptoms can appear slowly after 7–10 days. Thus, the disease could unintentionally spread among the community [34]. The B.1.524 lineage appeared to be the major contributor during the third wave of the pandemic, with D614G and A701V mutations noticed in the viral spike protein [35]. Cases increased drastically in Sabah from June to September 2021. The wide distribution of the Delta variant: clade GK and AY lineage, as shown in Figure 3 and Figure 5, is the cause of the increase in case numbers in the community. The Delta variant entered Malaysia via travellers returning from countries with high COVID-19 burdens, such as the United Kingdom, Sri Lanka and India [36,37]. As of June 2021, more than 80% of the SARS-CoV-2 genomes sequenced were dominated by the Delta variant, which was found to have a higher infectious viral load and longer duration of infectious viral shedding, leading to a high peak in the number of daily COVID-19 cases in Malaysia including Sabah [38]. Unfortunately, Malaysia has experienced one of the highest rates of COVID-19 infection in Southeast Asia, with deaths per capita surpassing two populous countries: India and Indonesia [39]. During this phase, the Malaysian government geared up the National COVID-19 Immunisation Programme and was recognised as having one of the fastest COVID-19 vaccination rates in the world, distributing an average of 400,000 doses per day to the adult population [40]. The highest number of deaths (1058) in Sabah was recorded on September 2021, and the full vaccination status of the adult population in Sabah failed to reach 50% during the period [41]. In early 2022, a similar trend of increasing cases was encountered in which new Omicron subvariants were detected in the community circulation [42]. It was evident that the number of cases seemed to increase with each variant switching. The sporadic infections in Sabah have become a trend in almost all districts. Since vaccination in children under 11 years was ongoing, numerous clusters involving educational institutions contributed to the number of cases [43,44]. Most cases were asymptomatic, and the number of deaths in Sabah was relatively low compared to when the Delta variant was prevalent. In April 2022, Malaysia announced a nationwide transition to the endemic phase by lifting all restrictions. However, the continuous tracking of SARS-CoV-2 variants is warranted to avoid another unnecessary outbreak among local communities that could trigger new waves of COVID-19 infections nationwide and subsequently worldwide. In this study, 583 SARS-CoV-2 genomes were sequenced and analysed along with other publicly available sequences to understand and timely monitor Sabah’s emerging variants and evolutionary patterns. Based on the collection period, epidemiological analysis of the early sequenced SARS-CoV-2 genome determined the O clade (March 2020), and the remaining sequences were in the G clade (October and December 2020). Hence, the L and GR clades were absent in the Sabah, as the L clade was dominant in the early pandemic (January 2020) and the GR clade around July 2020. Across the country, L clade was first identified among the country’s first three confirmed COVID-19 cases, and were similar to strains isolated from the Wuhan outbreak in December 2019. Furthermore, the S clade genome is infrequent (n = 4) in Malaysia and absent in Sabah, which was originally prevalent in the Americas and Oceania during the early pandemic [45]. Only two samples, defined as V clade from Malaysia, appeared in mid-January 2020. Next, clade G and GH and GR subclades were detected around February 2020. Since then, an increase in G, GH, and GR genomes has been noted in Europe and North America, dominating the GISAID database. However, Asia started recording a hike in all the clades a month after the European appearances. Over time, the L, V, and S clades gradually disappeared and were replaced by the G clade and its derivatives, GH and GR. In Sabah, clades GK and GRA were dominated during the fourth and fifth waves, respectively, in line with the global trend. The main reason that the majority of SARS-CoV-2 sequences in Sabah were GK and GRA clades was due to the sequencing efforts in Sabah being in full swing after June 2021 to support the national genomic surveillance of SARS-CoV-2 by tracking the VOCs in the country. This captured most clades in SARS-CoV-2 genomes from Sabah as GK and GRA.

The global molecular clock estimated 27.09 substitutions per year, and the data analysis of the current study revealed 28.37 substitutions per year in 2022 [46]. A slightly higher clock rate was recorded in Sabah, likely due to the lower number of sequences being analysed in this study compared to the global analysis. The time-scaled phylogeny revealed the origin of Sabah SARS-CoV-2, most probably from Indonesia, which was spread indirectly from Peninsula Malaysia in early March 2020. On a cautionary note, it should be pointed out that sampling may have been biased due to the relatively low number of samples being sequenced in Sabah during the early pandemic. In addition, the phylodynamic analysis showed exponential growth of SARS-CoV-2 in March 2020 as congruent with the virus introduction and spread into the local community prior to detection. The occurrence of a plateau in May 2020 resulted from the effect of nationwide lockdown and strict social distancing. Analysis of maximum genetic diversity in terms of Pangolin lineages showed only B.6.1 lineage in Sabah during the second outbreak of COVID-19. This lineage was associated with clusters related to Tabligh and immigration depot and was originally predominant in India [24]. Despite B.1 and B.1.1 (G614) being the first lineages detected in Malaysia, they were transmitted no faster than another D614, co-circulating mutated lineage during the second wave. In addition, B.1.1.354 was detected in multiple clusters involving the northern region of West Malaysia during July and August 2020. However, this may not rule out the absence of B.1 and B.1.1.354 lineages in Sabah, given the small number of samples sequenced during the allotted period. Therefore, the status of circulating lineages from April to September 2020 is scarce in Sabah. During the third wave in Malaysia, B.1.524 was assigned as the primary causative variant responsible for the higher number of COVID-19 cases in the country, where the outbreak was initiated by clusters from Sabah [5]. The origin of the B.1.524 lineage in Asia was first traced back to the Philippines. Hence the possible entry of this lineage into the country could be from Sabah, located close to the Philippines [33]. Therefore, B.1.524 lineage was documented in Sabah in October and December 2020, and the lineage was linked with other major clusters, including Damanlela construction, Perigi and Teratai clusters [47]. This lineage was displaced by a new Delta variant B.1.617.2 during the early fourth epidemic wave, and 53 of its descended lineages were identified subsequently in Malaysia. During the late phase of the fourth epidemic wave, the AY lineage outcompeted the B.1.617.2 lineage and encountered different dominant lineages in West and East Malaysia. The AY.23 lineage was dominant in Sabah and Sarawak as of August 20221 compared to the AY.59 and AY.79 lineages in West Malaysia during the June to December surveillance period 2021. The emergence of the AY.23 lineage in East Malaysia may be related to migrants from Kalimantan, Indonesia, crossing the land border and facilitating the transmission of the AY.23 lineage, a dominant lineage in Indonesia during the same surveillance period [48]. Notably, the unique AU.2 lineage was exclusively detected only in Sarawak during the fourth wave of the pandemic and was not detected in Sabah and other states from West Malaysia. This strengthens the notion that geographical location, the origin of the strains, and COVID-19 disease management by individual states could result in discrepancies in the type of circulating lineages in a country. This was illustrated by the distinct lineages between West Malaysia (AY.59 and AY.79), Sabah (AY.23) and Sarawak (AU.2) in the period spanning from June to December 2021, where the government prohibited inter-state travel under phases 1 and 11 of the National Recovery Plan. The introduction of the Omicron variant into Sabah eventually stopped the dominance of the Delta variant, which circulated for a longer time in the community (Figure 3A). The first Omicron variant, B.1.1, was detected in Sabah in February 2022, a few months after the detection from West Malaysia. Travellers visiting Middle Eastern countries introduced the first imported Omicron cases in Malaysia [49]. After replacing the Delta variant, Omicron caused a sharp increase in the number of COVID-19 cases, but the severity of the disease was reduced compared to the previous variant [24]. Sabah recorded an accumulation of fewer cases during the fifth wave of COVID-19 infection compared to the fourth wave of infection. A significant reduction in hospitalizations and patients requiring ventilator support was observed nationwide during the Omicron circulation [24]. The BA.1.1 (January to April 2022) and BA.2 (April to December 2022) sublineages of the Omicron variant were replaced by another BA.2 descendent sublineage XBB in Sabah from November 2022. Since then, sublineages of XBB.1 were the dominant and actively circulating in Sabah until 31 December 2022.

The average mutation rate per sample was recorded in this study as 56.9 (most of the sequences from 2022), while other studies recorded 7.23 and 17.7 in 2020 and 2021, relative to the reference genome [45,50]. This demonstrated the high rate of mutation per sample over time. Most mutations in all VOCs occurred in the receptor-binding domain (RBD), followed by the spike protein’s N-terminal domain (NTD). In this study, 23 and 12 notable mutations were recorded in RBD and NTD regions, respectively, and some mutations were shared in more than one VOC with different frequencies. Since most of the samples from Sabah were sequenced during the fourth and fifth waves of the pandemic, the mutational analysis in this study was mainly focused on Delta and Omicron variants. The most common mutations found in both West Malaysia and Sabah were a nonsynonymous mutation in the S protein, Spike_D614G, and a missense mutation of a segment of ORF1ab encoding the RNA-dependent RNA polymerase, NSP12_P323L, with a frequency of 99% in both regions [49]. Interestingly, about 99.5% of a synonymous mutation in the predicted phosphodiesterase papain-like proteinase (NSP3) mutation, F106F, was detected in West Malaysia and Sarawak but was absent in the Sabah samples. This could be explained by the timeline of each mutation occurrence, where the mutation F106F was recorded among the Beta variants in late 2021.

## 5. Conclusions

In conclusion, we describe the epidemiological landscape of COVID-19 in Sabah, Malaysia, considering the driving factors of multiple waves of the COVID-19 pandemic in the country. Different SARS-CoV-2 variants have been reported to dominate during each successive wave of infection and exhibit no sign of slowing down. Determining the variants using whole genome sequencing is crucial for tracking and tracing transmission, and monitoring the evolutionary pattern of the virus in a real-time setting could better discern the dynamics of a pandemic. The distribution of clades and lineages over a two-year period (2020–2022) provides valuable insight into the diversity and evolution of SARS-CoV-2 variants in Sabah. Moreover, highlighting the real-time status of the COVID-19 pandemic in East Malaysia could help to achieve the objectives of the national SARS-CoV-2 genomic surveillance programme. The data generated from this study add context to the global data and international genomics consortiums. In-depth characterization of SARS-CoV-2 mutations in Sabah could elucidate the movement of specific mutational patterns between individuals and across geographic areas. Therefore, continuous tracking of molecular epidemiology is needed to better understand viral pathogenicity, diagnosis and treatment of COVID-19 to contain further outbreaks.

## Data Availability

All data presented in this study are provided in the paper as main figures.
